# Cellular SUMO-specific proteases regulate HAdV-C5 E1B-55K SUMOylation and virus-induced cell transformation

**DOI:** 10.3389/fcimb.2024.1484241

**Published:** 2024-09-27

**Authors:** Wing-Hang Ip, Marie Fiedler, Britta Gornott, Malte Morische, Luca D. Bertzbach, Thomas Dobner

**Affiliations:** Department of Viral Transformation, Leibniz Institute of Virology (LIV), Hamburg, Germany

**Keywords:** deSUMOylation, E4orf6, human adenovirus, oncogenes, post-translational modification, sentrin-specific protease (SENP), small ubiquitin-like modifier (SUMO), viral transformation

## Abstract

Various viral proteins are post-translationally modified by SUMO-conjugation during the human adenovirus (HAdV) replication cycle. This modification leads to diverse consequences for target proteins as it influences their intracellular localization or cell transformation capabilities. SUMOylated HAdV proteins include the multifunctional oncoprotein E1B-55K. Our previous research, along with that of others, has demonstrated a substantial influence of yet another adenoviral oncoprotein, E4orf6, on E1B-55K SUMOylation levels. Protein SUMOylation can be reversed by cellular sentrin/SUMO-specific proteases (SENPs). In this study, we investigated the interaction of E1B-55K with cellular SENPs to understand deSUMOylation activities and their consequences for cell transformation mediated by this adenoviral oncoprotein. We show that E1B-55K interacts with and is deSUMOylated by SENP 1, independently of E4orf6. Consistent with these results, we found that SENP 1 prevents E1A/E1B-dependent focus formation in rodent cells. We anticipate these findings to be the groundwork for future studies on adenovirus-host interactions, the mechanisms that underlie E1B-55K SUMOylation, as well as the role of this major adenoviral oncoprotein in HAdV-mediated cell transformation.

## 1 Introduction

Post-translational modifications (PTMs) remarkably diversify proteins in terms of protein maturation, function, localization, and adaptability (Sims and Reinberg, 2008; Beltrao et al., 2013). PTMs include a wide variety of protein modifications such as phosphorylation and acetylation, but also conjugation with proteins as in ubiquitination and SUMOylation pathways (Hochstrasser, 2009; Beltrao et al., 2013). Importantly, PTMs are reversible and play key roles in virus infections, including infections with human adenoviruses (HAdVs) (Bergström Lind et al., 2013; Sohn et al., 2015; Wilson, 2017).

Research on PTMs of HAdV proteins, especially those by HAdV species C type 5 (HAdV-C5) has strongly improved our general understanding of the HAdV replication cycle and cellular transformation. Especially research on SUMOylation (that is, covalent conjugation of small ubiquitin-like modifier (SUMO) proteins) of HAdV proteins or HAdV-mediated SUMOylation of host cell proteins has recently gained momentum and revealed important new aspects of viral replication, virus-host cell interactions and virus-induced cell transformation (Everett et al., 2013; Sohn and Hearing, 2016; Fan et al., 2022; Ip et al., 2023b). Interestingly, SUMOylation of the multifunctional adenoviral large E1B protein E1B-55K determines its intracellular localization and consequently its transforming capacity, which occurs through the cooperation of adenoviral E1A proteins with E1B-55K as well as with the adenovirus E4 region-encoded oncoproteins E4orf6, E4orf3, and E4orf3/4 (Endter and Dobner, 2004; Kolbe et al., 2022; Ip et al., 2023a; Bertzbach et al., 2024). A single amino acid mutation at lysine 104 (K104) of the E1B-55K SUMO-conjugation motif has been shown to fully abrogate E1B-55K SUMOylation accompanied by a dramatic decrease in transformation efficiencies of rodent cells, suggesting that SUMOylation and thus, intracellular localization of E1B-55K affect HAdV-mediated cell transformation (Endter et al., 2001; Kolbe et al., 2022; von Stromberg et al., 2023). In addition, a recently reported single amino acid mutation at a lysine in very close proximity to the main E1B-55K SUMO conjugation motif, lysine 101 (K101), has been shown to substantially increase E1B-55K SUMOylation accompanied by elevated nuclear localization of the protein (Kolbe et al., 2022). Consequences of the K101R mutation on HAdV-mediated cell transformation, however, remain largely elusive.

Likewise, a protein that is encoded in the E4 transcriptional unit is also known to interfere with the cellular SUMOylation machinery. The multifunctional protein E4orf6 acts as a regulatory factor for E1B-55K SUMOylation as it has been shown that E4orf6 deletion mutants have increased E1B-55K SUMOylation levels (Lethbridge et al., 2003). This E4orf6-mediated reduction of E1B-55K SUMO levels is intriguing and we could recently show that their interaction is required for this process (Fiedler et al., 2022).

SUMOylation is facilitated by cellular (and viral) SUMO E3 ligases and constantly reversed by sentrin/SUMO-specific proteases (SENPs), a.k.a. SUMO proteases (Lowrey et al., 2017). To date, six human SUMO-deconjugating SENP isoforms are described, which are termed SENP 1, 2, 3, 5, 6, and 7. All six isoforms can proteolytically process and thereby “activate” SUMO proteins. Additionally, they also catalyze the deconjugation of SUMO proteins from substrate proteins (Yeh et al., 2000; Mukhopadhyay and Dasso, 2007; Hickey et al., 2012). These SENPs are classified into three groups depending on their sequences, evolutionary relationship, substrate proteins, and localization in the cell: SENP 1 and 2, SENP 3 and 5, and SENP 6 and 7 (Hickey et al., 2012; Kunz et al., 2018). Notably, SENP 4 is considered a pseudogene (Drag and Salvesen, 2008).

In this study, we assessed the interaction of HAdV-C5 E1B-55K with cellular SENPs and investigated specific deSUMOylation activities. First, we show that E1B-55K interacts with SENP 1 and is deSUMOylated by SENP 1. Next, we determined the impact of SENP 1 in cooperation with E1A and E1B proteins on cell transformation and our data reveal an effective inhibition of E1A/E1B-dependent transformation by SENP 1-mediated deSUMOylation of E1B-55K. Finally, we demonstrate that this SENP 1-mediated reduction of E1B-55K SUMO levels occurs independently of E4orf6 expression.

## 2 Materials and methods

### 2.1 Cells and culture conditions

H1299 cells (ATCC no. CRL-5803; American Type Culture Collection; Manassas, VA, USA) and baby rat kidney (BRK) cells (Nevels et al., 1997; Speiseder et al., 2014) were kept in incubators at 37°C in a 5% CO_2_ atmosphere and maintained in Dulbecco’s modified Eagle medium (DMEM; Gibco; Carlsbad, CA, USA) with 10% fetal bovine serum (PAN Biotech; Aidenbach, Germany) and antibiotics (100 U penicillin/100 µg streptomycin per ml, PAN Biotech). All cell lines were regularly monitored for mycoplasma contamination.

### 2.2 Plasmids and transient transfections

All recombinant plasmids were generated by site-directed mutagenesis using the vectors pcDNA3 and pCI-FLAG. SENP-encoding plasmids (kindly provided by Dr. Stefan Müller), plasmids encoding 6x His SUMO 3 (Tatham et al., 2009), SUMO 3 Q90P (this work, forward primer 5’-GAT GTG TTC CAA CAG CCG ACG GGA GGT TAG-3’, reverse primer 5’-CTA ACC TCC CGT CGG CTG TTG GAA CAC ATC-3’), HAdV-C5 E1B-55K (Nevels et al., 2001), the E1B-55K K101R and K104R mutants (Kolbe et al., 2022) and the HAdV-C5 E4orf6 (Querido et al., 2001) were used for transient polyethylenimine (PEI; Polysciences; Warrington, PA, USA) transfections (Schreiner et al., 2010).

### 2.3 Protein analyses

Transfected cell pellets were lysed on ice using radioimmunoprecipitation assay (RIPA) buffer (50 mM Tris/HCl pH 8, 150 mM NaCl, 5 mM EDTA, 1% P-40, 0.1% SDS, and 0.5% sodium deoxycholate). The lysates were then sonicated and centrifuged to remove cell debris. To investigate protein-protein interactions, one part of the resulting supernatant was immunoprecipitated for 2 h at 4°C followed by centrifugation at 600 × g for 5 min at 4°C exactly as described previously (Berscheminski et al., 2013). The remaining supernatant was boiled at 95°C for 5 minutes in 5% Laemmli buffer (input). Samples were stored at -20°C until further analyses by sodium dodecyl sulfate-polyacrylamide gel electrophoresis (SDS PAGE) and immunoblotting.

For nickel-nitrilotriacetic acid (Ni-NTA) SUMO pulldown analyses, cells were harvested, washed with pre-cooled PBS, and lysed in guanidine hydrochloride (GuHCl) buffer (6 M Guanidinium-HCl, 10 mM Tris and 100 mM sodium phosphate buffer pH 8.0). His-SUMO modified proteins were coupled to the Ni-NTA agarose beads (Thermo Scientific) by incubation overnight at 4°C. Next, His-SUMO conjugates coupled to the Ni-NTA agarose were pelleted by centrifugation, washed, and His-SUMO conjugated proteins were eluted from the beads and stored at -20°C until further use (Tatham et al., 2009).

For immunoblotting, SDS PAGE-separated proteins were transferred to nitrocellulose membranes with a pore size of 45 μm (GE Healthcare; Chicago, IL, USA) via wet electroblotting using the TransBlot Electrophoretic Transfer Cell System (BioRad; Hercules, CA, USA). Next, membranes were incubated in 5% non-fat dry milk-PBS solution for 1 h at 4°C to saturate non-specific antibody binding sites. The membranes were then washed 3 times with PBS-tween and incubated with the respective primary antibody at 4°C (**Table 1**). After 3 hours of incubation, membranes were washed and incubated with the respective horseradish peroxidase (HRP)-conjugated secondary antibody for 2 h at 4°C. Membranes were washed again and proteins were visualized on X-ray films using the SuperSignal West Pico Chemiluminescent Substrate (Thermo Scientific; Waltham, MA, USA) according to the manufacturer’s instructions. X-ray films were developed using the GBX Developer (Kodak; Rochester, NY, USA) and digitalized.

**Table 1:** Antibodies.

Antibody	Concentration	Company or reference
Mouse mAb AC-15 (β-actin)	1:5,000	Sigma-Aldrich (St. Louis, MO, USA)
Mouse mAb 6x His (SUMO)	1:5,000	Clontech (Mountain View, CA, USA)
Mouse mAb M2 (FLAG)	1:2,000	Sigma-Aldrich
Mouse mAb 2A6 (E1B-55K)	1:10	(Sarnow et al., 1982)
Mouse mAb RSA3 (E4orf6)	1:10	(Marton et al., 1990)
HRP α-mouse IgG	1:10,000	Jackson (West Grove, PA, USA)

### 2.4 Transformation assays

BRK cells were seeded in 6-well plates and transfected with the respective plasmids with calcium phosphate following the manufacturer’s protocol (ProFection Mammalian Transfection System, Promega; Madison, WI, USA). Transfected cells were cultivated for 4–8 weeks with weekly media changes until multilayered cell accumulations (foci) were visible. Foci were visualized by crystal violet staining and quantified in the same way as described previously (Speiseder et al., 2014). These experiments were repeated as four independent replicates.

### 2.5 Statistical analyses

Statistical analyses were performed using Graph-Pad Prism v9 (GraphPad Software, Inc.; La Jolla, CA, USA). A one-way analysis of variance with Dunnett’s multiple comparisons test was used to analyze focus formation in BRK cells and data were considered significant if p values were ≤ 0.05. Data are presented as means and error bars indicate standard deviations (SDs).

## 3 Results

### 3.1 E1B-55K interacts with and is deSUMOylated by SENP 1

**Figure 1:**
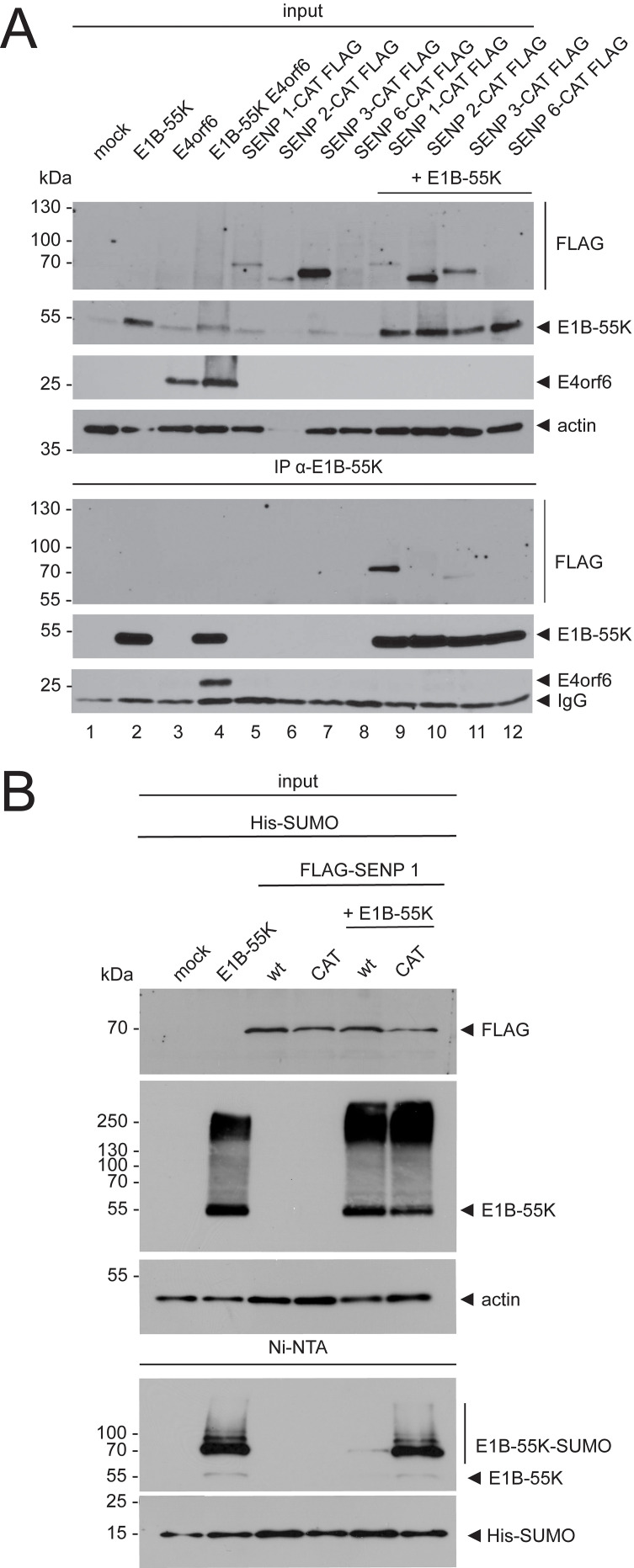
E1B-55K interacts with and is deSUMOylated by SENP 1. **(A)** H1299 cells were transfected with plasmids that encode the indicated catalytically inactive FLAG-tagged SENP isoforms and E1B-55K. E1B-55K and E4orf6 alone, or in combination were included as controls. Immunoprecipitations of E1B-55K were performed with mAb 2A6 (E1B-55K), precipitates were resolved by SDS PAGE and visualized by immunoblotting. Co-precipitated proteins and input levels of total cell lysates were detected using mAb 2A6 (E1B-55K), mAb RSA3 (E4orf6), mAb M2 (FLAG), and mAb AC-15 (actin). **(B)** H1299 cells were transiently transfected with E1B-55K and FLAG-tagged SENP 1 or SENP 1-CAT-expressing plasmids. His-SUMO modified proteins were precipitated by Ni-NTA pulldown and total cell lysates were prepared. Precipitates and protein inputs were separated according to their molecular weight by SDS PAGE and visualized by immunoblotting. For specific protein detection, mAb 2A6 (E1B-55K), mAb M2 (FLAG), mAb 6x His (SUMO), and mAb AC-15 (actin) were used. Molecular weights in kDa are indicated on the left and the corresponding proteins are labeled on the right. Displayed are blots that are representative of >3 separate experiments.

To test if E1B-55K interacts with SENPs, we performed co-immunoprecipitation (co-IP) assays using plasmids expressing E1B-55K and catalytically inactive (CAT) FLAG-tagged SENP isoforms from all three SENP groups (SENPs 1, 2, 3, and 6). These catalytically inactive SENPs were used to elongate interactions between SENPs and E1B-55K as deSUMOylation is a quick process and consequently, interactions between SENPs and their targets are rather unstable (Kunz et al., 2018). E1B-55K precipitation and subsequent FLAG-staining revealed an interaction between SENP 1-CAT and E1B-55K, indicated by a SENP 1-CAT-specific band at 70 kDa (**Figure 1A**). Moreover, a rather faint band in SENP 3-CAT/E1B-55K co-transfections indicates their interaction. These results suggest that E1B-55K could be targeted for deSUMOylation by SENP 1 and 3, although SENP 1 likely is the main SENP that deSUMOylates E1B-55K, given the much stronger staining (**Figure 1A**). In fact, SENP 6-CAT expression was very weak, and steady-state concentrations were not detectable (**Figure 1A**). On the other hand, SENP 1-CAT steady-state concentrations were also rather low, while the co-IP showed a solid enrichment of the protein (**Figure 1A**). These observations suggest that an interaction between E1B-55K and SENP6 is unlikely, but we have to note that it is difficult to make a definitive statement regarding the binding of E1B-55K to SENP 6. Collectively, however, our investigations on the interactions of E1B-55K with SENPs identified E1B-55K as a specific interaction partner of SENPs 1 and 3 (**Figure 1A**).

To assess the deSUMOylation of E1B-55K by SENP 1, we performed Ni-NTA pulldown analyses using H1299 cells that overexpress SUMO. We transfected these cells with different combinations of plasmids that express E1B-55K, SENP 1, and a catalytically inactive SENP 1 (SENP 1-CAT) (**Figure 1B**). Here, the His-SUMO pulldowns showed that SENP 1 reduced E1B-55K SUMO modification. At the same time, co-expression of the catalytically inactive mutant SENP 1-CAT did not affect SUMO modification of E1B-55K (**Figure 1B**).

### 3.2 SENP 1 overexpression results in efficient inhibition of E1A/E1B-dependent cell transformation

**Figure 2:**
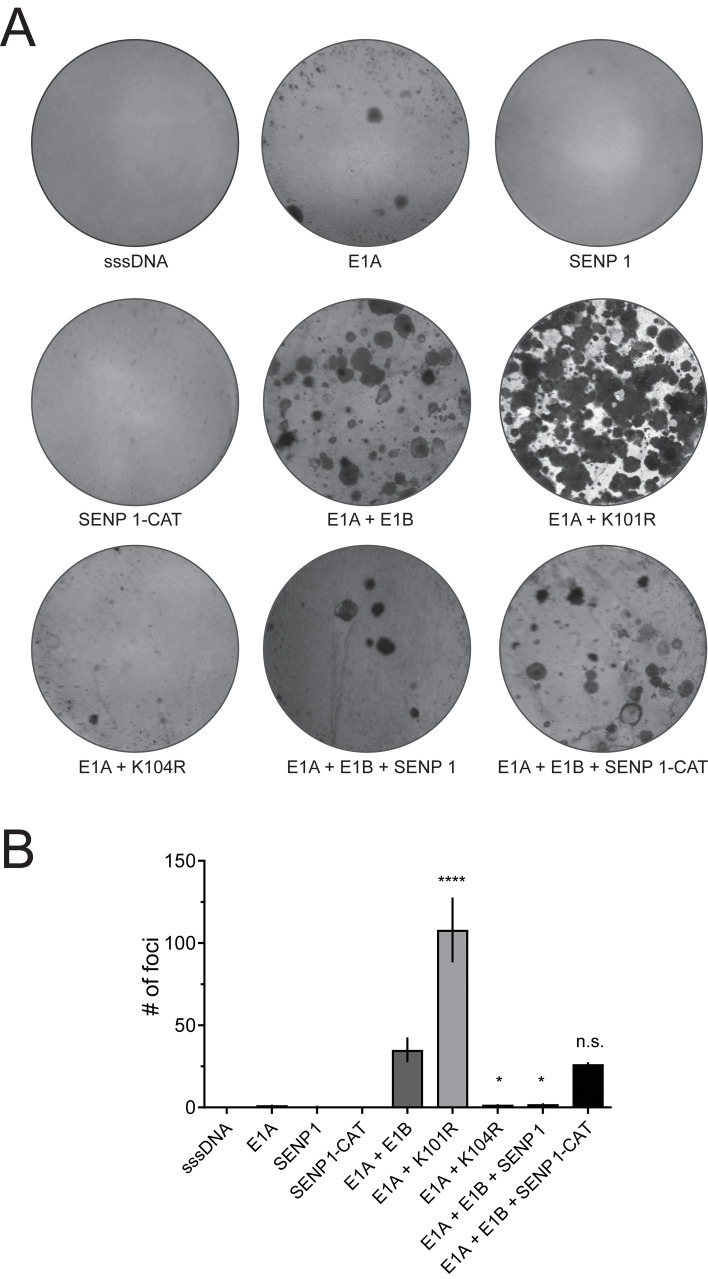
SENP 1 averts cell transformation, which is highly increased in HAdV-C5 E1B-55K K101R-transfected BRK cells. **(A)** BRK cells were transfected with plasmids encoding E1A alone or in combination with wt E1B-55K, K101R, or K104R. SENP 1 or SENP 1-CAT were transfected alone or together with E1A and E1B as indicated. sssDNA was transfected as DNA carrier. The cells were kept in culture for eight weeks before the plates were fixed and stained with a crystal violet solution. The illustration shows one representative experiment of four repeated assays. **(B)** Foci of each plate were quantified and plotted, and the error bars show the SD. Asterisks indicate significant differences (p-values were obtained from a one-way ANOVA with a Dunnett’s multiple comparisons test (*p ≤ 0.05, ****p ≤ 0.0001), comparing data to ‘E1A + E1B’). sssDNA: sheared salmon sperm DNA.

To investigate the effect of SENP 1 on E1A/E1B-dependent transformation of rodent cells *in vitro*, we transfected BRK cells with E1A and E1B-expressing plasmids alone or in combination with plasmids expressing active and inactive SENP 1. As controls for E1B-55K SUMOylation, either K104R (negative, mutation abrogates E1B-55K SUMOylation) or K101R (positive, mutation increases E1B-55K SUMOylation) (Kolbe et al., 2022) were transfected in combination with E1A. Our transformation experiments confirmed that E1A and E1B expression result in efficient transformation of BRK cells (**Figures 2A, B**). E1A and K101R co-transfections induced a remarkably increased transformation rate, while E1B-55K almost lost its transforming capabilities upon introduction of the K104R mutation (**Figure 2**). Co-transfections of E1A and E1B-expressing plasmids with SENP 1 revealed that expression of SENP 1 causes a significant decrease in the foci numbers, indicating that SENP 1 inhibits E1A- and E1B-dependent transformation of BRK cells through efficient deSUMOylation of E1B-55K (**Figure 2**). Concomitantly, co-transfections of E1A and E1B with the catalytically inactive SENP 1 (SENP 1-CAT) restored focus formation to wild-type (wt) levels (**Figure 2**). In sum, these data confirm that deSUMOylation of E1B-55K is mediated by SENP 1, leading to strongly reduced proliferation-promoting features of this adenoviral multifactorial oncoprotein.

### 3.3 SENP 1-mediated E1B-55K deSUMOylation is E4orf6-independent

**Figure 3:**
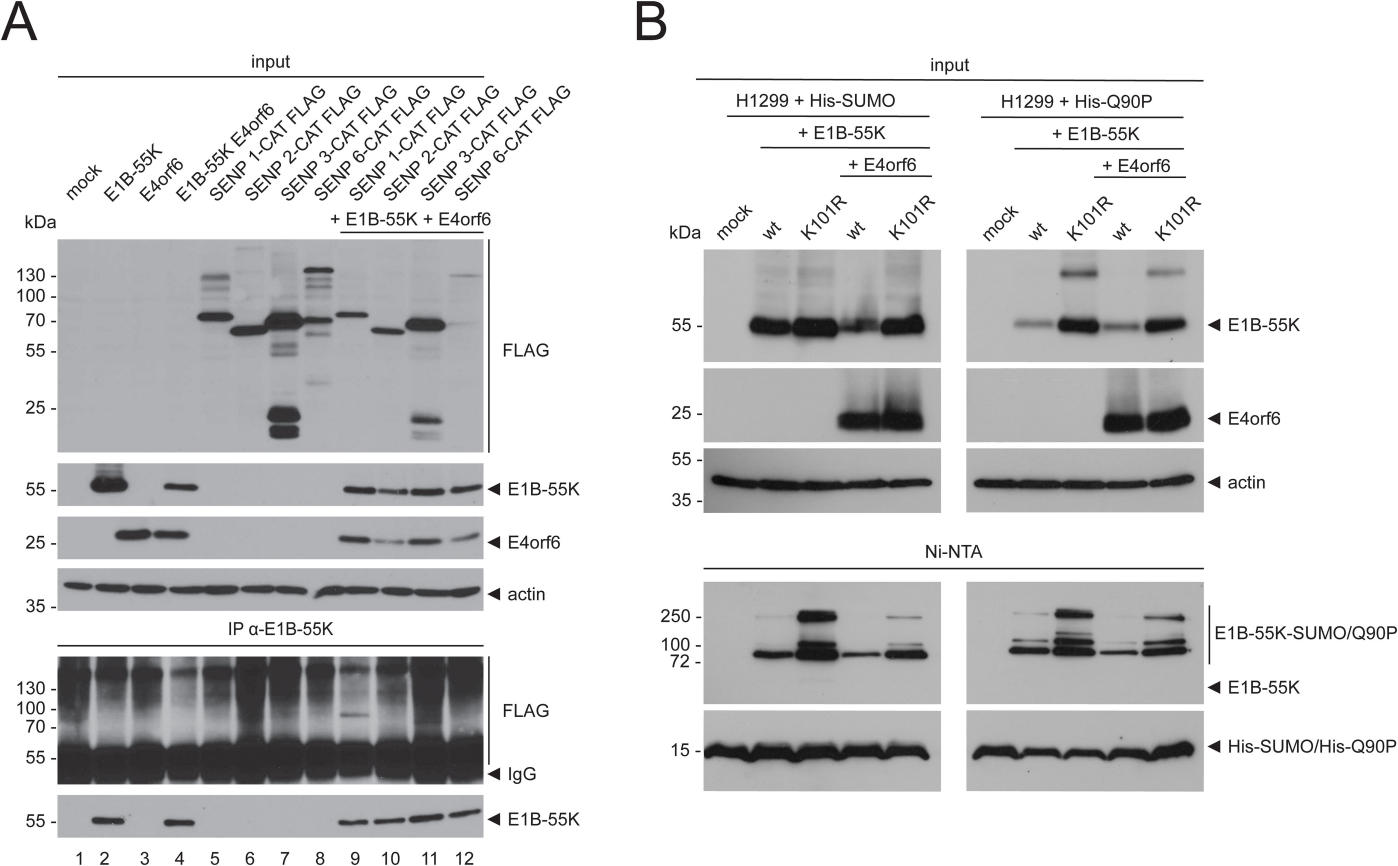
E1B-55K binds to SENP 1 and SENP 3 in the presence of E4orf6 and E4orf6 inhibits SUMO attachment to E1B-55K. **(A)** H1299 cells were transfected with the indicated catalytically inactive FLAG-tagged SENP isoforms, E4orf6 and E1B-55K-expressing plasmids. Immunoprecipitations of E1B-55K were performed using mAb 2A6 (E1B-55K). **(B)** H1299 cells were transiently transfected as indicated with plasmids encoding His-tagged SUMO, Q90P, wt E1B-55K, E1B-55K K101R, and E4orf6. His-SUMO modified proteins were precipitated by Ni-NTA pulldown. Co-precipitated proteins and input levels of total cell lysates were resolved by SDS PAGE and visualized by immunoblotting, using mAb 2A6 (E1B-55K), mAb RSA3 (E4orf6), mAb M2 (FLAG), mAb 6x His (SUMO) and mAb AC-15 (actin). Molecular weights in kDa are indicated on the left and the corresponding proteins are labeled on the right. Displayed are blots that are representative of >3 separate experiments.

To determine if E4orf6 influences the interaction of E1B-55K with SENPs, both proteins were transfected into H1299 cells in combination with the SENP CAT isoforms 1 to 3 and 6, followed by immunoprecipitation assays (**Figure 3A**). These experiments showed that the binding of E1B-55K to SENPs was not abrogated by E4orf6 because E1B-55K still efficiently bound to SENP 1-CAT and rather weakly to SENP 3-CAT both in the presence (**Figure 3A**) or absence of E4orf6 (**Figure 1**). Further, our results suggest that E4orf6 does not recruit additional SENPs for E1B-55K deSUMOylation. However, it cannot be excluded that E4orf6 deSUMOylates E1B-55K independently of SENP 1. To test this, we used a SUMO mutant that harbors a glutamine (Q) to proline (P) mutation at its SENP-cleavage site at position 90 (Q90P) leading to structural changes within SUMO that prevent deSUMOylation by SENPs (**Figure 3B**) (Owerbach et al., 2005; Bekes et al., 2011). Upon transfection with wt E1B-55K and the hyper-SUMOylation mutant K101R without and with E4orf6, and after subsequent Ni-NTA pulldowns, we found that co-transfection of E4orf6 resulted in reduced SUMO levels of E1B-55K and K101R (**Figure 3B**). Similar to wt SUMO, Q90P was conjugated to E1B-55K and K101R (**Figure 3B**), and Q90P-conjugation to E1B-55K and K101R were also decreased in presence of E4orf6 (**Figure 3B**). Thus, we confirmed that SENP 1 reduces SUMO on E1B-55K and demonstrate that this process can be, in part, compensated by E4orf6.

## 4 Discussion

The adenoviral E1B-55K oncoprotein is a key player in various processes during the viral replication cycle and for viral transformation (Blackford and Grand, 2009; Hidalgo et al., 2019). E1B-55K is SUMO-modified, a process that is negatively regulated by another adenoviral oncoprotein, E4orf6. While recent research focused on E1B-55K SUMOylation, its deSUMOylation through cellular proteins, i.e. the reversion by SUMO-specific proteases, remained elusive. In this report, we present SENP 1 as the protease that deSUMOylates E1B-55K and substantiate these data by a strong reduction of E1B-55K-mediated cell transformation upon SENP 1 overexpression.

We first demonstrated that E1B-55K interacts with and is deSUMOylated by SENP 1. These findings, consistent with previous observations showing the deSUMOylation of Kap1 by SENP 1 (Li et al., 2007; Bürck et al., 2016), support the hypothesis that functionally related protein groups are targeted by the same SENPs (Psakhye and Jentsch, 2012; Jentsch and Psakhye, 2013).

Next, we demonstrated that SENP 1 overexpression efficiently inhibits E1A/E1B-dependent cell transformation. It is well-established that E1A/E1B-induced transformation of rodent cells occurs through repression of p53-stimulated transcription, a characteristic of HAdVs (Yew and Berk, 1992; von Stromberg et al., 2023; Bertzbach et al., 2024). Our findings reveal that (i) SENP 1-mediated deSUMOylation of E1B-55K reduces its ability to form foci, and (ii) the high SUMOylation of the K101R variant is associated with a “gain-of-function,” leading to increased repression of p53-stimulated transcription (Kolbe et al., 2022; von Stromberg et al., 2023) and, consequently, increased focus formation in BRK cells.

Finally, our data show that E4orf6 can partially compensate for the SENP1-mediated deSUMOylation of E1B-55K. Interestingly, a recent report characterized the impact of E4orf6 on the expression of SENP 8. In contrast to the other SENPs, this cysteine protease is not involved in deSUMOylation but deconjugates the ubiquitin-like protein NEDD8 from target proteins. This deNEDDylase is downregulated by E4orf6, resulting in an increased NEDDylation of cullin 5, which in turn suppressed p53 degradation (Guo et al., 2019). Our data indicate that, in addition to ubiquitination, and NEDDylation, E4orf6 also plays a role in regulating another PTM: protein SUMOylation. These and our findings extend the list of E4orf6 functions (Ohman et al., 1993; Nordqvist et al., 1994; Dobner et al., 1996; Jayaram et al., 2008; Müller et al., 2012).

In addition, the interactions between E1B-55K and SENP 1 could possibly manipulate nuclear import and export processes to favor the transport of viral late mRNAs and late viral proteins (Woo and Berk, 2007; Blanchette et al., 2008). SENPs 1 and 2 have been shown to be involved in nuclear pore complex integrity (Hang and Dasso, 2002; Zhang et al., 2002; Goeres et al., 2011; Chow et al., 2014) and one could speculate that binding to viral proteins could alter their function and thereby modulate the functionality of the nuclear pore complex.

In sum, our results further link the SUMO-conjugation machinery with adenovirus-induced cell transformation and highlight new aspects of the interaction of adenoviral oncoproteins with regulatory host enzymes. Future work could include infection of SENP knockdown or knockout cells to further understand consequences of these interactions.

## Data Availability

The original contributions presented in the study are included in the article/supplementary material. Further inquiries can be directed to the corresponding author.
